# Visualization of Metabolic Interaction Networks in Microbial Communities Using VisANT 5.0

**DOI:** 10.1371/journal.pcbi.1004875

**Published:** 2016-04-15

**Authors:** Brian R. Granger, Yi-Chien Chang, Yan Wang, Charles DeLisi, Daniel Segrè, Zhenjun Hu

**Affiliations:** 1 Bioinformatics Program, Boston University, Boston, Massachusetts, United States of America; 2 Center for Advanced Genomic Technology, Boston University, Boston, Massachusetts, United States of America; 3 Department of Biomedical Engineering, Boston University, Boston, Massachusetts, United States of America; 4 Department of Biology, Boston University, Boston, Massachusetts, United States of America; UCSD, UNITED STATES

## Abstract

The complexity of metabolic networks in microbial communities poses an unresolved visualization and interpretation challenge. We address this challenge in the newly expanded version of a software tool for the analysis of biological networks, VisANT 5.0. We focus in particular on facilitating the visual exploration of metabolic interaction between microbes in a community, e.g. as predicted by COMETS (Computation of Microbial Ecosystems in Time and Space), a dynamic stoichiometric modeling framework. Using VisANT’s unique metagraph implementation, we show how one can use VisANT 5.0 to explore different time-dependent ecosystem-level metabolic networks. In particular, we analyze the metabolic interaction network between two bacteria previously shown to display an obligate cross-feeding interdependency. In addition, we illustrate how a putative minimal gut microbiome community could be represented in our framework, making it possible to highlight interactions across multiple coexisting species. We envisage that the “symbiotic layout” of VisANT can be employed as a general tool for the analysis of metabolism in complex microbial communities as well as heterogeneous human tissues. VisANT is freely available at: http://visant.bu.edu and COMETS at http://comets.bu.edu.

This is a *PLOS Computational Biology* Software paper.

## Introduction

Metabolism comprises a complex network of biochemical reactions that guarantee a supply of energy and building blocks to every living cell. More broadly, however, metabolism can be viewed as a multicellular or ecosystem-level phenomenon, encompassing not only the biochemical transformations occurring within each cell, but also the exchange of molecules across different cells or organisms. Indeed, most microbial life takes place in complex multi-species microbial communities whose dynamics is highly dependent on metabolite-mediated microbe-microbe and microbe-environment interactions. Through such interactions, soil and ocean communities participate in the global cycling of oxygen, carbon and nitrogen [1,2], while human-associated communities can alter the balance between health and disease [3,4]. Thus, understanding how the genome-encoded functionalities of individual species affect global community interactions and dynamics has important environmental and biomedical applications. In parallel to metagenomic sequencing approaches, the systems biology community has been developing tools to simulate the complete metabolic network activity of individual microbes and natural or engineered microbial communities [5–10]. These tools are focused on a predictive, quantitative understanding of metabolite-mediated interactions between species using extensions of flux balance analysis (FBA) [11]. A flexible implementation of ecosystem-level metabolism is at the core of a software platform named COMETS (Computation of Microbial Ecosystems in Time and Space) [12]. COMETS is a free, open-source tool that predicts metabolic rates across different interacting species at different time points in a spatially structured environment, by integrating dynamic FBA (dFBA, [13]) with diffusion equations. COMETS has been validated experimentally on small synthetic microbial ecosystems [12]. It can include many different species, enabling simulations of how ecosystem-level interaction networks may emerge as a consequence of individual organisms’ genome-scale metabolism. Additional implementations of dFBA-based simulations of ecosystem-level metabolism have been recently successfully applied to other small communities ([14,15]).

The opportunity to scale genome-level models to simulate complex communities poses new analysis and visualization challenges. One major challenge is the implementation of hierarchical representations of these systems and the outcomes of model simulations. Ideally, all the details of intracellular networks would be maintained, but at the same time researchers would be able to zoom out to obtain an ecosystem-level view of the whole community, with a major emphasis on the network of metabolites exchanged between species. A number of tools have been developed for analyzing and/or visualizing metabolic network and fluxes [16–27], with layout solutions ranging from novel automated algorithms to reproduction of standard textbook-like topologies. To the best of our knowledge, however, no tools have been specifically designed to help study genome-scale metabolic networks at the ecosystem level.

Here we present an approach that addresses the challenge of extracting data from genome-scale models, and visualizing the ensuing ecosystem-level network, with numerous potential applications to the study of the human microbiome and other microbial communities. Our approach was developed within release 5.0 of VisANT, a workbench for the integrative analysis of biological networks. In comparison to other tools [16–27], VisANT provides unique features, such as exploratory navigation to walk through the interactions [28] and metagraph capability for multi-scale visualization with integrated context information (such as protein complex, GO category and disease classification). The latter is achieved through the presence of metanodes—special nodes that allow embedding of subnetworks [29–32], with individual nodes potentially belonging to more than one metanode. Moreover, VisANT’s visualization capabilities are supported by a knowledge base, in which nodes and edges are associated with information about the corresponding biological entities, and are connected to additional resources, such as the KEGG Reaction and KEGG Compound databases[33].

New features in VisANT 5.0 are aimed at facilitating the visual exploration of metabolite exchange and competition between microbes in a community. The main features of VisANT 5.0 that make this possible are: 1) Metagraph-based multi-model visualization, where each metabolic network model is encapsulated by an expandable metanode. 2) New layout algorithms and color schemes to classify metabolites as mediating different types of interactions between organisms (e.g. cross-feeding or competition); 3) Dynamic flux visualization to capture the time-dependent network variation and flux distribution, with the capability to export the changes as animated GIF files; 4) Comparison of the flux distribution of the community-level network under different conditions; 5) Exploratory navigation of reactions and metabolites; 6) Query of enzymes to facilitate the integration of signaling networks, regulatory networks, and expression data. 7) Capacity to edit metabolic networks, through navigation of built-in hierarchy of Enzyme Commission (EC) nomenclature.

## Design and Implementation

VisANT is a widely used tool for the building and analysis of biological networks. Initially focused on the interaction network between bio-molecules in its first release [28], VisANT gradually expanded its scope into the fields of systems biology, systems pharmacology and translational science with the support of metagraph capability [29–31,34] and integrated knowledge of diseases, therapies and drugs [32]. By embedding a subnetwork into a metanode, the metagraph capability not only provides the multi-scale visualization and interference of the network, but also allows the same data to be analyzed from different perspectives with appropriate network transformation functions [32]. Meanwhile, VisANT keeps records to allow users to trace back the original credits of the corresponding databases [33,35,36] while providing users the convenient assistance in network construction and analyses with the power of integrated knowledge [30,37,38].

### Input/output

**Fig 1:**
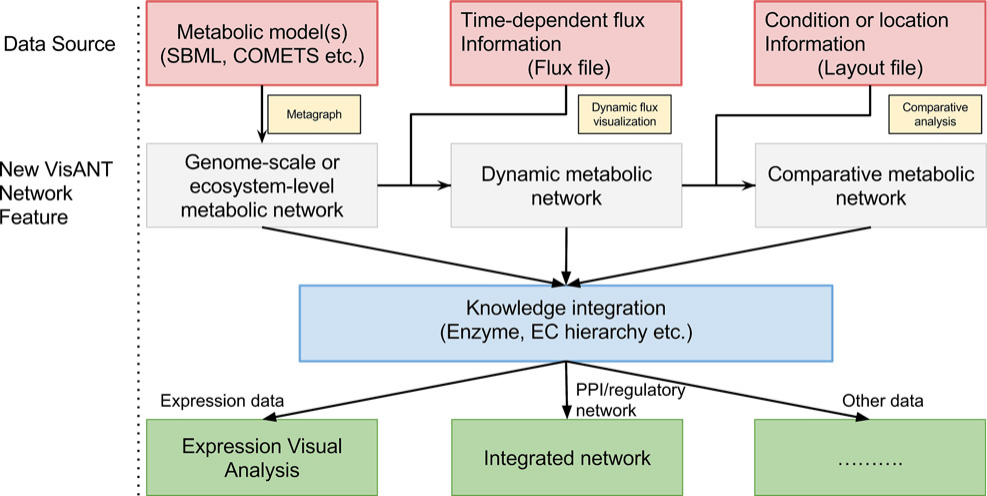
Workflow of visualization of metabolic networks and associated data. This chart describes the flow of data inputs and analyses in VisANT 5.0, with focus on functionalities which help interpret community-level models of microbial metabolism. VisANT 5.0 integrates three main data types (top pink layer) pertaining to ecosystem-level metabolism: (i) One or more stoichiometric models of metabolic networks for different organisms; (ii) Flux data for all reactions in all organisms at different time points; (iii) A layout file where spatial information of metabolic models is specified. Adding these different types of information on top of each other leads to visualization of different features of the network (gray layer). These networks can be combined with additional knowledge on the catalyzing enzymes, such as regulatory effects or protein-protein interaction edges (bottom layer, green).

As shown in Fig 1, the new functions in VisANT 5.0 are built around three main data types: (i) The network models, which can be uploaded from SBML (Systems Biology Markup Language) files [39–41], from COMETS input files, or from a tab-delimited edge-lists that includes the specification of node type; (ii) The flux matrix, containing information on the rates (or fluxes) of different reactions in different organisms at different time points. This flux matrix can be either uploaded from standard COMETS output files, or from plain text files, provided that the reaction IDs match the reactions IDs in the corresponding models; (iii) A layout file, if available, containing the abundance of different organisms and metabolites at different locations. Note that inputs (ii) and (iii) are optional, i.e. VisANT can visualize a metabolic network model irrespective of whether flux or spatial information is available. Moreover, VisANT can generate these interaction networks from generic files and is not limited to COMETS simulations (see User Manual, Page 5, S1 Text).

To further simplify the construction of a network from multiple models, VisANT adopts a Manifest file format comparable to that produced by COMETS, where multiple files of different types can be specified in one place, using either a local path or a URL. The resulting networks can be saved as a VisANT XML file (VisML) that preserves all the information for users to start a new analysis exactly from the point at which the network was saved. Networks can be exported as tab-delimited edge-lists, and saved as images or high-resolution SVG files. While in the current work we describe in detail only analyses performed using COMETS output files, the capacity of VisANT to represent community-level networks from other sources (e.g. SBML files generated from Model SEED [42]) is detailed in the User Manual–*Working with your own data*, at Page 5 (S1 Text).

### Metagraph-based visualization of metabolic reactions

VisANT 5.0 uses a standard bipartite graph representation of metabolic reactions, in which two types of nodes, metabolites and reactions, are connected through directed edges to visualize the product-substrate structure (S1 Fig). Furthermore, through the metagraph-based approach [34] VisANT can represent each reaction by a metanode with embedded enzyme nodes (S1C and S2 Figs). This approach preserves the advantages of a simple bipartite graph (when metanodes are collapsed) while at the same time holding the detailed enzyme protein information for each reaction (visible when metanodes are expanded). This feature is particularly useful for studies requiring the integration of metabolic networks with regulatory or signaling networks, as illustrated in S2 Fig.

### Data integration

One of VisANT’s main goals is to assist network analysis by integrating biological knowledge from different sources or layers of complexity. In this release of VisANT, we add integration of KEGG reaction and compound databases and the Enzyme Commission (EC) hierarchy. These new features not only provide easy exploration of the functional classification of enzymes, but also support the creation of reaction nodes with convenient drag & drop operations from the Hierarchy Explorer (left side of VisANT) to the main network panel (see User Manual—*Functions associated with built-in Enzyme Nomenclature* at Page 14, S1 Text). Keyword searching is available for EC hierarchy, providing indirect query of reactions based on functional descriptions.

### Visualization of ecosystem-level metabolic networks

**Fig 2:**
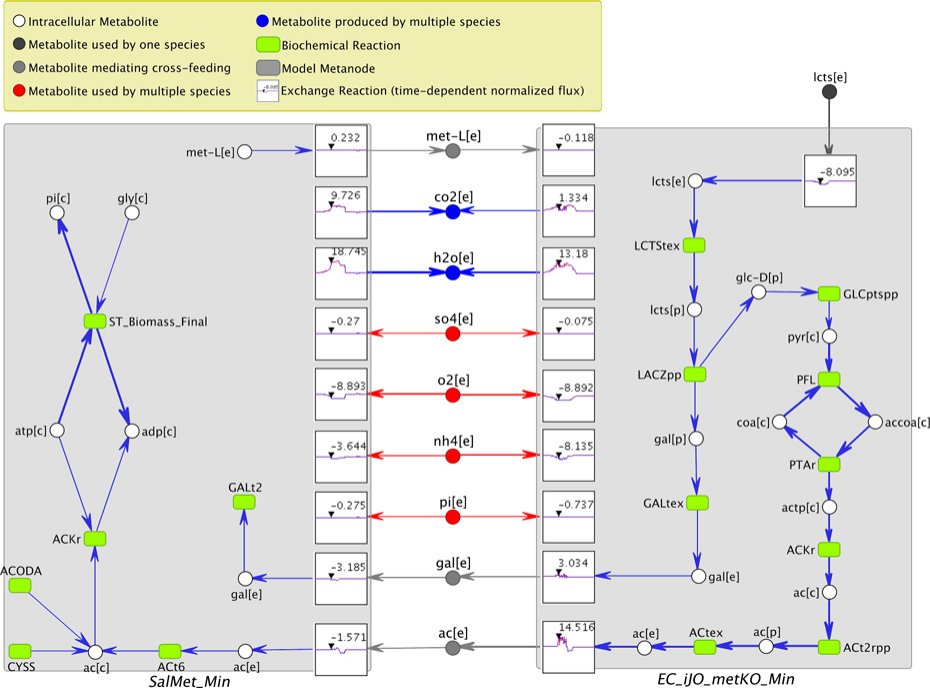
VisANT visualization of metabolic cross-feeding between two bacteria, using the new “Symbiotic Layout” functionality. This specific system is a previously evolved, obligate syntrophic consortium between a genetically modified *E*. *coli* strain which requires an external supply of methionine, and a *S*. *enterica* strain that cannot use the only carbon source available in the environment (lactose). The system was simulated with COMETS (Computation of Microbial Ecosystems in Time and Space), and represented in VisANT. For this case study we used a single spatial point (i.e. a 1 by 1 grid in COMETS), thus loading one grid point or the entire simulated COMETS grid are equivalent. Models are represented as expanded metanodes, exchange reactions are shown as nodes with (X,Y) graphs representing the flux through them throughout the simulated growth experiment, with an arrow denoting the current time step. Extracellular metabolite nodes are color coded based on the type of interaction they mediate: (i) Blue if it is secreted by both organisms; (ii) Red if it is consumed by both; (iii) Light gray if one model produces it and the other consumes it, and (iv) Dark gray if the metabolite is only associated with one model. *E*. *coli* can be seen here taking up lactose, and secreting acetate as a by-product. *S*. *enterica*, in turn, is able to grow using the acetate, and secretes methionine, which allows *E*. *coli* to continue to grow. Users can trace through the network by double-clicking a node to reveal connected nodes. This was used to trace lactose through the *E*. *coli* network, and to display some of the intracellular reactions adjacent to the exchange fluxes in *S*. *enterica*. In order to make the differences in fluxes more apparent for the reactions that mediate interactions between the two species, all the corresponding fluxes were mapped logarithmically onto the edge weights, using the "Rescale Selected Edges Logarithmically” function (see User Manual–*Rescale Selected Edges Logarithmically* at Page 10, S1 Text).

**Fig 3:**
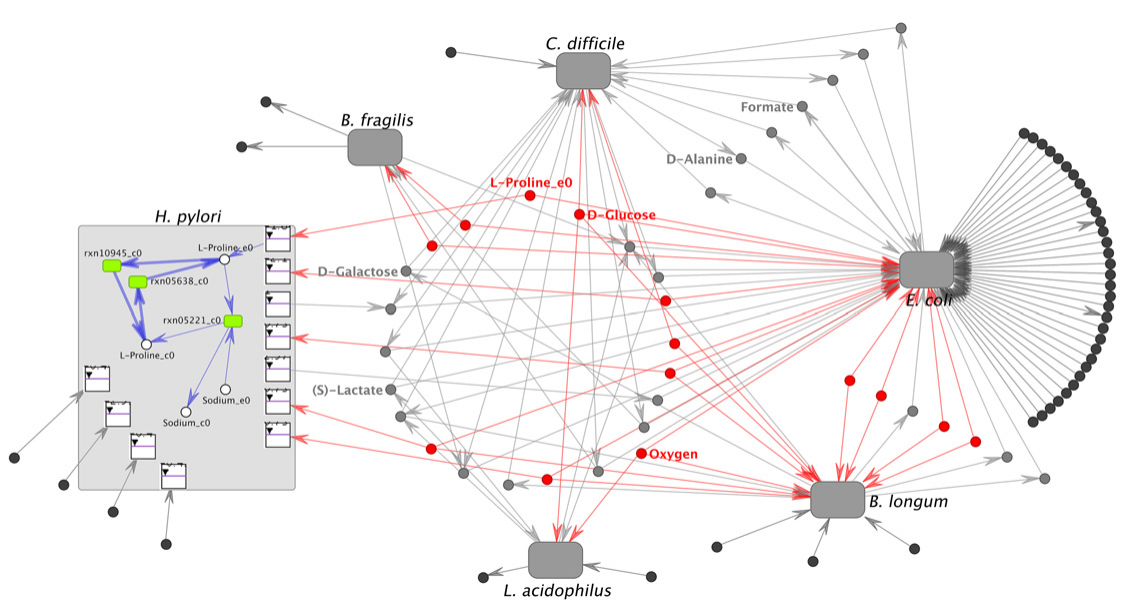
Metabolic exchange in a microbial ecosystem. Visualization of metabolic exchange between many organisms in a system is achieved through VisANT's multi-organism layout. The six organisms shown here are microbes selected because of their roles and abundance in the human gut. Metabolic flux was determined through a COMETS simulation involving all six microbes in a minimal D-glucose media. Each model is represented by a metanode, and in the center of the models are nutrients in the media that interact with the models. Nodes are color coded by their syntrophic influence, gray being nutrients involved in a potential syntrophy, red being nutrients which the microbes may be competing over, blue being nutrients which are produced by more than one organism, but consumed by none, and dark gray being nutrients which only have one model interacting with them. The network is simplified by hiding metabolite nodes that are not transported in/out by any model at this time point (normally displayed in white color), as well as metabolites not useful for biological interpretation of interactions, including biomass subcomponents (Protein_biosynthesis_e0, RNA_transcription_e0, and DNA_replication_e0) and highly connected ubiquitous metabolites (H2O_e0, H+_e0, Orthophosphate_e0). Minor manual rearrangement was conducted for the expanded network to improve clarity. Five of the six model metanodes are collapsed for clarity, but *H*. *pylori* is shown expanded, displaying the environmental exchange reactions. Nutrients of interest, Oxygen, D-Glucose, D-Galactose, (S)-Lactate, and Formate, have been labeled. Part of the L-Proline intracellular pathway has been expanded to exemplify VisANT’s capabilities.

One of the main new features of VisANT 5.0 is the implementation of functions specifically designed to facilitate the visualization of the network of metabolite-mediated interactions between microbial species in a community, or different cell types in a tissue. Our “symbiotic network” function is made possible by the metagraph network representation. Metabolic networks for individual organisms are represented as distinct bipartite graphs, where one type of node represents reactions, and the other type of node represents metabolites, as described above. While in the current demonstration of the multi-species network we do not take advantage of the capacity of reaction nodes to hold enzyme information (S2C Fig), such information can in principle be queried against the VisANT database for supported organisms. The whole set of reaction and metabolite nodes for each cell or organism’s network is encapsulated by a metanode. The only exceptions are metabolites being exchanged between cells/organisms or with the environment. Such metabolites are duplicated outside of individual organisms’ metanodes, representing their capacity to serve as environmental mediators of interactions. Thus, multiple metabolic models can be linked to each other through metabolites that are either secreted or imported by the different species present in the same community (Fig 2). Metanodes of individual models can be collapsed, making it convenient to focus on the overall community structure and interaction (Fig 3). By default, the symbiotic layout displays only exchange reactions and transported metabolites. However, users can easily expand and explore specific portions of intracellular pathways of interest (see S1 Video), or choose to display the complete intracellular metabolic network.

One potential source of metabolic models and flux information which VisANT can utilize is the COMETS platform. The output of COMETS simulations includes flux solution vectors for each metabolic model in each location at each time point. COMETS output also includes time-dependent abundance of any extracellular (i.e. environmental) metabolite. The huge size of the multi-organism metabolic networks poses a great visualization challenge. We focused mainly on the development of functions that would help interpret the metabolic exchange (syntrophy) or the competition for common resources between cells/organisms. Metabolic network sizes may vary widely, based on the specific setup and biological questions being asked. The metabolic model of *E*. *coli* [43], when represented in VisANT, amounts to a network of 4,713 nodes, comprised of 1,805 metabolites, 2,583 reactions, 324 environmentally exchanged metabolites and one model metanode. These nodes are connected together by a total of 10,831 edges. Since microbial community simulations involve two or more metabolic models, the total network size grows quickly. For example, the network of six organisms shown in Fig 3 involves a total of 12,815 nodes and 28,749 edges. Multiple layout algorithms (Circle, Spoke, Spring Embedded Relaxing etc.) are available in VisANT. However, due to the nature and the complexity of the community-level metabolic network, none of these layouts would be able to automatically reduce the network complexity and help in the interpretation of the inter-species interactions. Therefore, in VisANT 5.0, we implemented a layout algorithm, named Symbiotic Layout, which draws the ecosystem-level network with a special emphasis on those reactions and metabolites involved in inter-species interactions. This layout is designed to reduce the network complexity, and provide an effective description of ecological interactions between species in a community, mediated by syntrophy and competition for common metabolites.

An example of a two-species microbial consortium is shown in Fig 2. Each stoichiometric model is represented as a metanode (in its expanded form). Metabolites exchanged with the environment are shown around the outside of the model metanodes, and connected via exchange reaction nodes. If both models connect to the same environmental metabolite, that metabolite is placed in between the two organisms. Otherwise, extracellular metabolites are placed on the external side of the model to which they belong. Metabolites may play different roles for different models in the community. A simple color scheme helps emphasize the different roles that environmental metabolites play within the community (Fig 2), mediating different types of interactions. Most notably, this representation makes it easy to view metabolites for which the two organisms compete, and metabolites that may mediate a cross-feeding interaction, i.e. are discarded by one species, and used as nutrient by the other. If the microbial community involves more than two organism models, VisANT uses a multi-organism layout. In this layout, model metanodes are arranged in a circle, and shared metabolites are gathered in the middle (Fig 3). These environmental metabolites are color-coded as above, so as to highlight their role as mediators of interactions. Note that since the fluxes may be dynamically changing throughout dFBA/COMETS simulations, the color of each metabolite may change over time, reflecting the corresponding type of interaction.

### Dynamic flux visualization

By default, VisANT shows all potential pathways as defined in the metabolic models and all possible exchanges defined in the ecosystem. When flux data are supplied, VisANT projects the flux distribution onto the metabolic network, adjusting each arrow to reflect the appropriate directionality of the reaction flow. By default, the edge thickness (rescaled between 0 and 1) is proportional to the intensity of the flux. The user can however choose to map the fluxes onto edge thicknesses logarithmically, to enhance visibility of fluxes that span multiple orders of magnitude (see User Manual–*Rescale Selected Edges Logarithmically* in page 10 for more details (S1 Text), and Fig 2 for an example). If the flux information is time-dependent, VisANT adds dynamic aspects to the generated metabolic network. This is achieved in multiple ways. A slider allows the user to view flux data throughout the whole network at any desired time points, with the option of automatically cycling through all time points. The dynamic flux visualization can also be exported as an animated GIF file. In addition, the flux of transport reactions can be visualized as a special larger node that contains a flux vs. time plot (Figs 2 and 3) with a small cursor pointing to the current time point.

### Comparative analysis

VisANT 5.0 supports comparative visualization of flux distributions in a community-level network across different conditions, and locations (when spatial information is supplied), with no limit to the number of states compared. When performing a comparative visualization, each edge is divided into several segments with different colors representing the flux of corresponding conditions (or locations), and the thickness of each segment is proportional to the amount of flux. Alternatively, for the comparison between two states, it is possible to scale the thickness of each arrow to reflect the difference between the fluxes under the two different conditions/locations. An example case study for the comparative analysis is available in S3 Fig.

## Results

### Case Study 1—Visualization of cross-feeding in a 2-species microbial consortium

In the first case study (S1 Dataset), we examined metabolic networks and metabolite-mediated interactions between an *E*. *coli* and a *S*. *enterica* strains previously designed and evolved to form an obligate syntrophic consortium [12,44]. The *E*. *coli* strain had been genetically modified such that it cannot synthesize methionine. *S*. *enterica* cannot use lactose as a carbon source. In the evolved consortium, the two organisms can survive on lactose only in the presence of each other, by exchanging methionine and a carbon source (likely acetate) respectively. This consortium has been studied experimentally and simulated computationally in previous work [12]. We used a COMETS simulation of this system to generate files to be imported in VisANT, with the goals of visualizing and corroborating previous insight on the metabolic exchange between the two organisms.

To investigate COMETS data for this consortium using VisANT, a user can open a COMETS Manifest file under the “Symbiosis” menu. The user can select a particular point, or visualize the metabolic activity of the whole system, averaged over the different spatial locations. VisANT will process the model and flux files designated by the COMETS Manifest file, and automatically provide a multi-species layout. By default the network is initially displayed with no overlaid flux values. The layout consists of two metanodes, representing the two organisms. In our case, flux information was additionally loaded (as specified by the Manifest file), and can be viewed by advancing the slider at the top of the screen. In the default symbiotic layout, only exchange reactions are initially displayed. Additional reactions/metabolites can be displayed by double clicking on nodes whose connectivity is considered of interest. In order to facilitate the interpretation of the results, one can remove low intensity fluxes (by setting a weight cutoff threshold) and hide highly connected metabolites (listed in a file specified by the Manifest file). These operations should be followed by a recalculation of the symbiotic layout. For this case study, we set the lower weight cutoff threshold for rescaled fluxes to 10^−5^ and removed highly connected cofactors (e.g., ATP, ADP, NAD(H), NADP(H)). The final VisANT VisML file representing this network is available as Supplementary File 1.

As seen in Fig 2, this sequence of steps leads to a community-level network that is amenable to biological interpretation. Three of the environmental metabolite nodes (in gray) correspond to metabolites that mediate mutualistic (i.e. cross-feeding) interactions. These include acetate (ac[e]) and methionine (met-L[e]), thought to be essential in the syntrophic exchange between these two organisms. Additionally, we can see that lactose (lcts[e]) is utilized only by *E*. *coli*, which in turn secretes the acetate usable by *S*. *enterica*. Curiously, at the particular time point analyzed, the exchange of galactose (gal[e]) also seems significant, and constitutes a novel testable prediction. Using VisANT’s ability to trace metabolites through the network, we can see that galactose is produced in *E*. *coli*, as a necessary consequence of lactose breakdown through β-galactosidase. While the glucose produced by this reaction proceeds through glycolysis, the galactose is predicted to be secreted, and utilized by *S*. *enterica*.

### Case Study 2—Visualization of metabolic interactions in a multi-species microbial community

For this second case study (S2 Dataset), we interrogated a multi-species microbial community and visualized the main flux exchanges between the organisms. The organisms were selected to represent some of the most abundant microbes in the human gut [45]. Models for these organisms were obtained (as gap-filled stoichiometric models, [46]) from the DOE Kbase (http://kbase.us/) [47], converted to COMETS models, and run all together in a simulation with a minimal D-glucose medium (containing traces of all other metabolites that are transportable in the different models, see S1 Table).

The user can visualize a simulation with more than two species by opening from VisANT the COMETS Manifest file exactly as described in Case Study 1. In this case, we load the Manifest file for a simulation involving six organisms, and have VisANT automatically arrange them in the Symbiosis Layout (with a flux visualization threshold of 10^−4^). This time we get the six model metanodes in a circle, surrounding all metabolites that interact with more than one model (Fig 3). As we advance the slider, we can see how the interactions change over time. Here we show the network at about one fifth of the simulation (time step 6). The final VisANT VisML file representing this network is available as Supplementary File 2. Importantly, this type of community analysis is not restricted to COMETS-generated data, and can be implemented by loading multiple model files (e.g. as SBML), and flux data (e.g. as flat files, see also User Manual–*Flux File* at Page 3, S1 Text).

The community-level network of human intestine-associated bacteria displayed in Fig 3 reflects some known information about these organisms, as well as potential limitations of the reconstructed networks and modeling framework. Most organisms are predicted to use glucose as a main carbon source, though for some organisms (*B*. *fragilis* and *H*. *pylori*) other carbon sources seem dominant. For example, in *H*. *pylori*, the model predicts a significant uptake of proline, which has been reported to be important for *H*. *pylori* gut colonization[48]. An interesting predicted syntrophic cross-feeding interaction is the one mediated by lactate, which is secreted by *L*. *acidophilus* and utilized by *C*. *difficile* and *E*. *coli*. In turn, *C*. *difficile* is predicted to utilize formate, which *E*. *coli* secretes as a by-product. This seems plausible since *C*. *difficile* is known to have a formate dehydrogenase enzyme [49]. Another intriguing environmentally exchanged metabolite is galactose, produced by *B*. *longum* and utilized by *C*. *difficile* and *E*. *coli*. Some evidence for this type of activity by *B*. *longum* has recently been presented in the literature [50]. The detailed step-by-step instructions for both case studies can be found in S2 Text, Test data and running instructions.

## Availability and Future Directions

VisANT 5.0, in conjunction with COMETS dFBA simulations of microbial consortia, was able to corroborate results from a well-studied syntrophic exchange, and to provide nontrivial insight into possible metabolite-mediated interactions in a putative complex multi-organism community. As long as our future understanding of gene functions catches up with our capacity to sequence genomes and build models [51], similar analyses of natural and artificial microbial communities could become a powerful tool for biomedicine, metabolic engineering, and biogeochemistry. VisANT’s unique metagraph implementation not only enables convenient approaches to investigate microbial community interaction either at a bird’s eye view or at great detail, but also provides great potential for further studies through integrated knowledge on regulation and other functional genomics studies. While the focus of this work has been on the integration of computationally predicted metabolite exchange and flux data, the symbiosis layout could be readily used to visualize similar experimental data that may become available in the future. Finally, although here we have only explored examples of interactions between bacteria in microbial communities, the same tools and visualization options are easily applicable to analyzing host-pathogen interactions[52,53], as well as interactions between different cell types in normal or cancerous human tissues[54,55]. VisANT and its source code are freely available at: http://visant.bu.edu and COMETS at http://comets.bu.edu.

## Supporting Information

S1 Table D-glucose medium composition for COMETS simulations. (XLSX)

S1 Dataset Data files for case study 1, zipped directory. (ZIP)

S2 Dataset Data files for case study 2, zipped directory. (ZIP)

S1 Fig Comparison of representations of metabolic reactions. (DOCX)

S2 Fig Metabolic network with integrated regulatory or signaling interactions. (DOCX)

S3 Fig Comparative analysis of the flux at the center vs. periphery of a colony. (DOCX)

S1 Text VisANT 5.0 user manual. (PDF)

S2 Text Test data and running instructions. (PDF)

S1 Video Exploratory navigation on the intracellular reactions and metabolites. (MP4)
