# Supplementary material for: Visualization of Metabolic Interaction Networks in Microbial Communities Using VisANT 5.0
Source: PLoS Comput Biol. 2016 Apr 15;12(4):e1004875. doi: 10.1371/journal.pcbi.1004875 (PMC4833320; doi:10.1371/journal.pcbi.1004875)
Supplement: S1 Fig — (DOCX) [file pcbi.1004875.s004.docx]

**
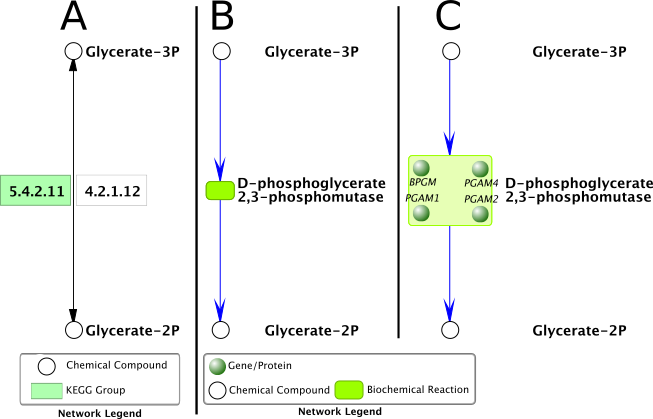
**

**Figure S1 - Comparison of representations of metabolic reactions**

A) In this representation (from KEGG pathways) a reaction is depicted as a line that connects metabolites to each other. This line is labeled by the enzyme name. B) VisANT uses an alternative representation, i.e., a bipartite graph, in which two types of nodes, metabolites and reactions, are connected through directed edges to visualize the product-substrate structure of the transformation. This representation, employed in VisANT 5.0, can be further enriched by the introduction of metanodes. C) When a reaction is represented by a metanode, the specific enzyme proteins associated with that reaction can be embedded in the metanode, enabling integration with other data related with such proteins. Note that in the current VisANT representation, information about reversibility (present in A) is not shown. This makes it possible to appropriately visualize reactants and products in the stoichiometric matrix, or (once flux values are uploaded) to show the actual direction of the metabolic flow.
