# Supplementary material for: Visualization of Metabolic Interaction Networks in Microbial Communities Using VisANT 5.0
Source: PLoS Comput Biol. 2016 Apr 15;12(4):e1004875. doi: 10.1371/journal.pcbi.1004875 (PMC4833320; doi:10.1371/journal.pcbi.1004875)
Supplement: S2 Fig — (DOCX) [file pcbi.1004875.s005.docx]

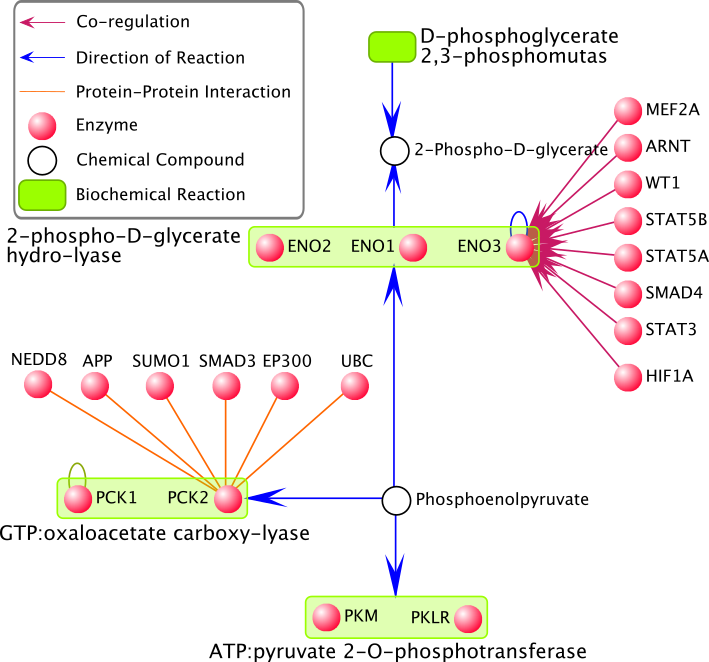


**Figure S2 – Metabolic network with integrated regulatory or signaling interactions**

The metagraph-based visualization schema allows the convenient integration of regulation and protein-protein interactions. Here the reactions 2-phospho-D-glycerate hydro-lyase, GTP:oxaloacetate carboxy-lyase, and ATP:pyruvate 2-O-phosphotransferase, have been annotated with genes that encode for enzymes that can perform these reactions. The ENO3 gene has been annotated with predicted co-regulated genes (MEF2A, ARNT, WT1, STAT5B, STAT5A, SMAD4, STAT3, HIF1A)(46). For the GTP:oxaloacetate carboxy-lyase reaction, protein-protein interactions of PCK2 are shown (NEDD8, APP, SUM01, SMAD3, EP300, UBC). The green loop at PCK1 and the blue loop at ENO3 indicate auto-regulation.
