# Supplementary material for: Visualization of Metabolic Interaction Networks in Microbial Communities Using VisANT 5.0
Source: PLoS Comput Biol. 2016 Apr 15;12(4):e1004875. doi: 10.1371/journal.pcbi.1004875 (PMC4833320; doi:10.1371/journal.pcbi.1004875)
Supplement: S3 Fig — (DOCX) [file pcbi.1004875.s006.docx]

**Case Study - Comparative Analysis**


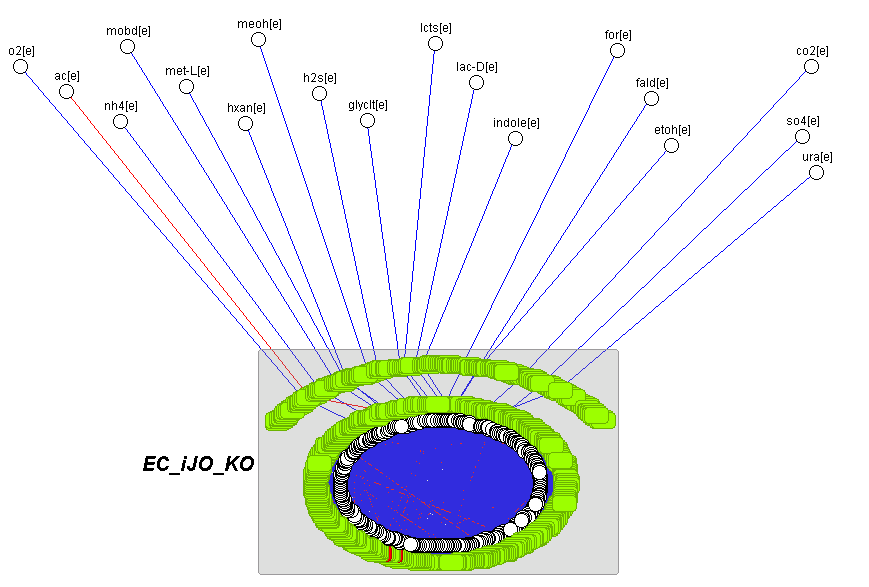


**Figure S3 – Comparative analysis of the flux at the center vs. periphery of a colony.**

This figure illustrates the capacity of VisANT to compare two different flux states, and visualize the changes in a useful way. This specific example pertains to the same system analyzed in Case Study 1 and Figure 2, in which *E. coli* and *Salmonella* colonies exchange metabolites in order to survive and grow. The comparison displayed here is between *E. coli* growing in two different regions of a colony, i.e. in the center vs. the periphery after 90 h of simulated growth time. What we observed in previous simulations is that while *E. coli* initially secretes acetate, some portions of the colony seem to switch to re-using it at later times. In the comparative VisANT representation, exchange reactions that change in magnitude are displayed in blue, while reactions that change in direction of the flux are displayed in red. As expected, the acetate flow is reverted between center and periphery *E. coli* cells, reflecting the expected behavior. To produce this visualization, the user can go through the following steps: (i) select a COMETS manifest file for the desired simulation; (ii) at the prompt, select the location of the first flux state to be compared (in this case, a point in the center of the colony). (iii) go to Comparative analysis tools, and specify the location of the second state for comparison (in this case, a point at the periphery of the colony). (iv) Select the desired time point for the analysis using the slider bar. Note that the comparative analysis can also be performed with other types of files, and can compare networks differing in underlying parameters, rather than location. Detailed instructions on how to perform these analyses are included in the VisANT User Manual.
