# Supplementary material for: Visualization of Metabolic Interaction Networks in Microbial Communities Using VisANT 5.0
Source: PLoS Comput Biol. 2016 Apr 15;12(4):e1004875. doi: 10.1371/journal.pcbi.1004875 (PMC4833320; doi:10.1371/journal.pcbi.1004875)
Supplement: S1 Text — (PDF) [file pcbi.1004875.s007.pdf]

Metabolic Network

Files supported by VisANT

VisANT 5.0 supports the visualization of genome-scale and ecosystem-level metabolic networks. The formats invloved are listed below:

Manifest File (optional)

This file specifies all files required for building a metabolic network, especially for the networks involving multiple models. VisANT will load files and build a network accordingly. It is highly recommended to have a manifest file for your data. Two types of manifest files are supported: COMETS manifest file and VisANT FBA manifest file. Both are a simple text file which enumerates the files and attributes required for the data visualization. Each row indicates a property-file(s) or property-value(s) pair separated by a colon. When a property is associated with more than one file or value, the files or values must be separated by a single space. The first line of the VisANT FBA manifest file must be

Example

```
#!FBA_Manifest
ModelFileName: model_CSP.mod model_CSP.mod model_2.mod
ModelLabel: model1 model2 mode3
FluxFileName: flux.txt
```

```
1.   #!FBA_Manifest
2.   ModelFileName:
3.   .......
4.   .......
```

A sample file which will construct a eco-system level metabolic network from SBML files can be downloaded from here (http://visant.bu.edu/sample/symbio/manifest.txt)

Example

```
1.   #!FBA_Manifest
2.   ModelFileName:http://visant.bu.edu/sample/symbio/Ec_core_flux1.xml http://visant.bu.edu/sample/symbio/Ec_core_flux1.x
     ml http://visant.bu.edu/sample/symbio/Ec_core_flux1.xml
3.   ModelLabel: Eco_model-01 Eco_model-02 Eco_model-03
4.   FluxFileName: http://visant.bu.edu/sample/symbio/test_flux_03.txt
```

Notes: you can copy-and-paste the above code into “Your data” field and click “Add” to run it in VisANT

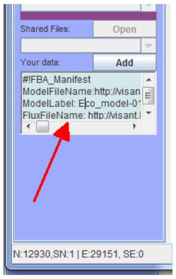

The required properties are

- ModelFileName
- ModelLabel

The optional properties are

- FluxFileName
- LayoutFileName (only required for the symbiosis result)
- UninterestingMetabolitesFileName

Property	Value
ModelFileName	A list of model files separated by a single space. VisANT accepts<br>• Simple file name: `model_CSP.mod`<br>• Absolute path: `/home/user1/models/model_CSP.mod`<br>• Relative path: `../../model_CSP.mod`<br>• URL: `http://yourdomain.edu/models/model_CSP.mod`
ModelLabel	A list of model names separated by a single space. A model name must be unique and the order of the names here must match the order of files that you specified in the ModelFileName.
FluxFileName	This file specifies all files required for building a metabolic network, especially for the networks involving multiple models. VisANT will load files and build a network accordingly. It is highly recommended to have a manifest file for your data. Two types of manifest files are supported: COMETS manifest file and VisANT FBA manifest file. Both are a simple text file which enumerates the files and attributes required for the data visualization. Each row indicates a property-file(s) or property-value(s) pair separated by a colon. When a property is associated with more than one file or value, the files or values must be separated by a single space. The first line of the VisANT FBA manifest file must be

VisANT also supports formats specified by COMETS software. A COMETS manifest file is auto-created by COMETS and usually named as COMETS_manifest.txt, which can be directly imported into VisANT. Here is an example of the COMETS manifest file

```
1.   LayoutFileName: petri_dish_circles.txt
2.   ModelFileName: model_CSP.mod model_2.mod
3.   FluxFileName: flux_log_20140404155741.txt
```

Notes: be sure all file paths are correct.

Model file (required)

Model files define the metabolic models. VisANT supports two different file formats

SBML

Each SBML file only specifies one metabolic model, it is therefore highly recommended to use a manifest file if your network has more than one model. VisANT uses the JSBML library to process SBML files. In order to keep VisANT light-weight, this function is implemented on the server side. Therefore VisANT will need internet access to process SBML files.

About exchange metabolites/reactions in SBML
VisANT uses the `boundaryCondition` attribute on `species` to determine whether a chemical compound is an exchange metabolite or not. When a `species` is on the boundary (the `boundaryCondition` attribute is equal to `true`), this `species` will be labeled as an exchange metabolite and VisANT will place it outside the model metanode in the metabolite network. For more details about the `boundaryCondition`, please read:

- http://sbml.org/Documents/FAQ (http://sbml.org/Documents/FAQ#What_is_this_.22boundary_condition.22_business.3F)
- http://sbml.org/Software/libSBML/docs/java-api/org/sbml/libsbml/Species.html (http://sbml.org/Software/libSBML/docs/java-api/org/sbml/libsbml/Species.html)

COMETS Model File

When working with COMETS, we highly recommend use of the COMETS Model File format description (http://www.bu.edu/segrelab/comets-metabolic-model-files/ (http://www.bu.edu/segrelab/comets-metabolic-model-files/)) Scripts for converting COBRA and SBML formats into COMET Model format are present here: http://www.bu.edu/segrelab/comets-utility-scripts/ (http://www.bu.edu/segrelab/comets-utility-scripts/)

Layout file (optional)

VisANT supports COMET Layout Format a formal description of the Layout File format description can be found on the COMETS website (http://www.bu.edu/segrelab/comets-layout-file/ (http://www.bu.edu/segrelab/comets-layout-file/)). This file provides the basic information concerning the model world such as the grid size, extracellular metabolites etc. If a layout file does not exist, VisANT will use the information in the model files to render your data.

Flux file (optional)

VisANT supports two different file formats, a COMET style flux file and a simple tab-delimited text file. Again, when working with COMETS, we highly recommended using the COMET Flux file. Example are rpovided below.

COMET Flux file

2D

Example

```
fluxes{1}{16}{21}{1} = [0 0 -9.6E-6 -2.1E-2 0];
fluxes{2}{16}{21}{1} = [0 7E-6 0 7.3E-4 3.2E-6];
fluxes{3}{16}{122}{2} =[4.7E-8 1.6E-4 5E-2 6E-8 2.7E-9];
fluxes{3}{16}{122}{3} =[2E-3 5E-3 6.8E-8 5.7E-9 -7.6E-2];
```

```
fluxes{timeCycle}{Xlocation}{Ylocation}{model#} = [flux_of_reaction_1 flux_of_reaction_2 ... ];
```

3D

Example

```
fluxes{1}{16}{21}{1}{1} = [0 0 -9.6E-6 -2.1E-2 0];
fluxes{2}{16}{21}{2}{1} = [0 7E-6 0 7.3E-4 3.2E-6];
fluxes{2}{16}{71}{1}{2} = [-6E-9 0 0 5E-3 ];
fluxes{3}{16}{122}{2}{2} =[4.7E-8 1.6E-4 5E-2 6E-8];
fluxes{3}{16}{122}{1}{3} =[2E-3 5E-3 6.8E-8 5.7E-9 -7.6E-2];
```

```
fluxes{timeCycle}{Xlocation}{Ylocation}{Zlocation}{model#} = [flux_of_reaction_1 flux_of_reaction_2 ... ];
```

Tab delimited flux file:

A simple tab delimited text file which contains the flux values of reactions in a matrix format. Each row includes a model name (specified in the manifest file), a reaction name, x-coordinate, y-coordinate, z-coordinate and then a series of flux values separated by tabs.

Example

```
model_1 reaction_1	16	21	1	0	0	0
model_1 reaction_2	16	21	1	0	0	0
model_1 reaction_3	16	21	1	-9.6E-6	0	0
model_1 reaction_4	16	21	1	-2.1E-2	0	0
model_1 reaction_5	16	21	1	0	0	0
model_1 reaction_1	16	21	2	0	0	0
model_1 reaction_2	16	21	2	0	7E-6	0
model_1 reaction_3	16	21	2	0	0	0
model_1 reaction_4	16	21	2	0	7.3E-4	0
model_1 reaction_5	16	21	2	0	0	0
model_2 reaction_1	16	71	1	0	-6E-9	0
model_2 reaction_2	16	71	1	0	0	0
model_2 reaction_3	16	71	1	0	0	0
model_2 reaction_4	16	71	1	0	5E-3	0
model_2 reaction_1	16	122	2	0	0	4.7E-8
model_2 reaction_2	16	122	2	0	0	1.6E-4
model_2 reaction_3	16	122	2	0	0	5E-2
model_2 reaction_4	16	122	2	0	0	6E-8
```

A sampel file can be downloaded from here (http://visant.bu.edu/sample/symbio/test_flux_03.txt)

UninterestingMetabolitesFileName file (optional)

This file provide a simple list of metabolites which are highly connected or considered uninteresting to the user for whatever reason. A user can decide to hide or show them in the output network. The file format consists of one line per metabolite, each containing the name of the metabolite used in the model files.

Example

```
cpd00002
cpd00008
cpd00063
```

Visualization of metabolic networks in VisANT

VisANT 5.0 supports the visualization of genome-scale and ecosystem-level metabolic networks. Each metabolic model is encapsulated by an expandable metanode. Expanded model metanodes display the complete metabolic network of single organisms. Collapsing the organism metanodes allows the user to hide intracellular details, and view overall community structure and interaction.
Several file formats are supported (see: supported file formats). For example, you can build an ecosystem-level metabolic network by importing multiple SBML files or COMETS model files into VisANT. However, we highly recommend working with a manifest file. A manifest file is a plain text file which contains all required information for building a metabolic network. Users can assemble a manifest by himself/herself or use ones created by COMETS.
Different set of features will be enabled depending on the supplied files. Here is a complete list

Features	Required input file(s)
A. Visualization of a genome-scale metabolic network (without flux)	**Option 1**<br>• A manifest file<br>**Option 2**<br>• A model file

B. Visualization of a genome-scale metabolic network (with flux)	• A manifest file
C. Visualization of an ecosystem-level metabolic network (without flux)	**Option 1** • A manifest file **Option 2** • A layout file **Option 3** • Multiple model files
D. Visualization of an ecosystem-level metabolic network (with flux)	• A manifest file
Comparative Analysis	• A metabolic constructed by step (B) or (D) • The flux file(s)

Working with COMETS

By default, COMETS will not produce any output files. Be sure to enable 'Write flux log' in COMETS (see: http://www.bu.edu/segrelab/comets-e-coli-simulation-example/ (http://www.bu.edu/segrelab/comets-e-coli-simulation-example/)). A COMETS manifest (by default named COMETS_manifest.txt) will be automatically generated in the output folder after every simulation. To visualize the COMETS results in VisANT, simple import the COMETS manifest file into VisANT

Working with your own data.

The best way to import your own data into VisANT is to create a custom manifest file. Details of how to prepare a manifest file can be found in the supported file formats section. A user defined manifest file should work in the same way as a COMETS manifest file. Alternatively, you may import your files one by one to build your own metabolic network from scratch. For instance, you can import several SBML files to build a metabolic network of a community.

Opening a manifest file

To build a metabolic network from a manifest file

1. click [Symbiosis] -> [File] -> [Open a manifest file]

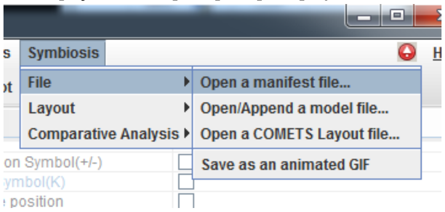

2. Select a manifest file
3. Decide if you want to visualize the flux distribution of the entire world in the simulation, or just the fluxes in a specific location. If you choose "Load the entire world', VisANT will average the flux distribution over the different spatial locations.

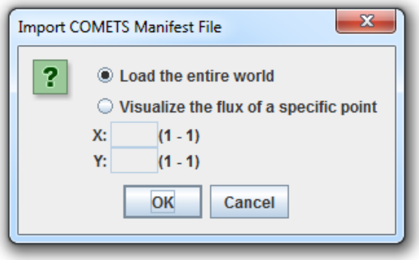

4. VisANT will process the layout file, model file(s) and flux file, then and project the flux distribution onto the metabolic network.

Opening/appending a model file

A metabolic model can be uploaded from a SBML file or a COMETS model file. To import a model file

1. click [Symbiosis] -> [File] -> [Open/Append a model file...]

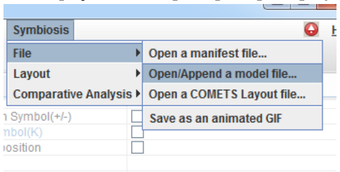

2. Select a model file (either a SBML file or a COMETS model file) from the dialog.
3. The metabolic model will be imported and encapsulated by a model metanode.

You can repeat the above procedure to ‘append’ additional metabolic models to your existing network. Each newly added metabolic model will form a model metanode and will receive a unique ID from VisANT.

Opening a COMETS Layout file

COMETS layout files specify the medium composition, the initial biomass condition and the spatial information of a model world. An ecosystem level metabolic network can be built from a COMETS layout file. To import a COMETS layout file

1. click [Symbiosis] -> [File] -> [Open a COMETS Layout file...]

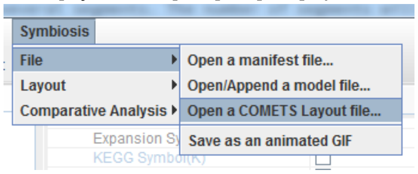

2. Select a COMETS layout file
3. VisANT will process the layout file and import the corresponding model files

The resulting network doesn’t have any flux information. To project the flux distribution into a metabolic network, you will have to begin with a manifest file.

Comparative analysis

The user can compare the flux distribution under different conditions or different locations in a spatial simulation using VisANT. When performing a comparative analysis, there are two ways to visualize the differences.

- By default, each edge will be divided into several segments. The number of segments will be equal to the number of conditions/locations. Each segment represents the flux in a corresponding condition or location, and the thickness of each segment is proportional to the amount of flux. The figure below shows an example where the red represents condition X and the blue presents condition Y. In this toy example, the flux in the condition X is greater than the flux in condition Y.

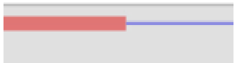

- When the number of conditions or locations the user wants to compare is 2, the user can choose to only show the difference of fluxes. In this type of data representation, the edge thickness will be proportional to the differences between the two fluxes in the different conditions (2nd condition/location - 1st condition/location).

To perform a comparative analysis

1. Build a metabolic network (with the flux distribution).
2. click [Symbiosis] -> [Comparative Analysis] -> [Compare with the fluxes in another location] or [Compare with the fluxes in diff. condition].

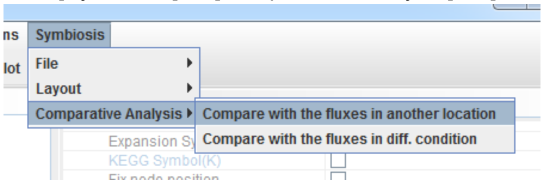

A. If you select [Compare with the flux in another location],specific the X, Y and Z-coordinates in the following dialog. Check [Show the differences of the fluxes] if you want VisANT to calculate the differences between two locations. Click the [Next, select a flux file] to go to the next step.

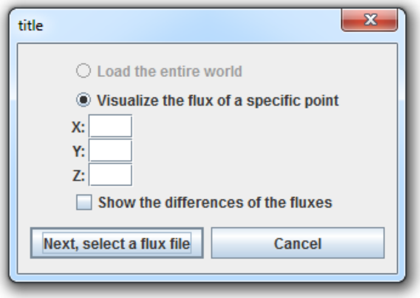

B. If you select [Compare with the flux in diff. condition], you won't be able to specific the X, Y and Z-coordinates in the following dialog. VisANT will compare the existing fluxes with the average fluxes over the different spatial locations in the second condition. Check [Show the differences of the fluxes] if you want VisANT to calculate the differences between two conditions. Click [Next, select a flux file] to go to the next step.

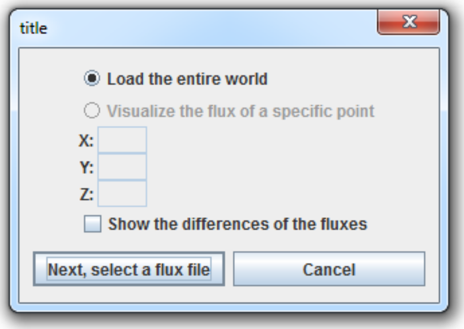

3. Select the flux file from the file choice dialog.
4. VisANT will process the flux data and build a comparative network.

Save the metabolic network as an animated GIF

You can export a dynamic metabolic network as an animated GIF file and share it online. To do so

1. Build a metabolic network.
2. Use the slider to specify the starting time point

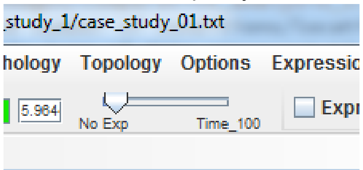

3. click [Symbiosis] -> [File] -> [Save as an animated GIF]

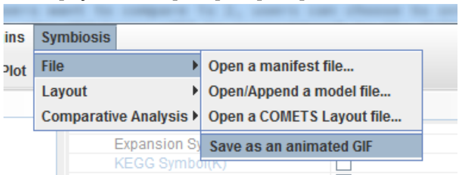

4. Specify the file name in the dialog and then click [Save] to save the file

---

Customize the color code schema

By default, when the flux distribution is available, VisANT will color the environmental metabolites based on the following rules

- Red: the metabolite is consumed by all models interacting with it.
- Blue: the metabolite is a by-product which is secreted by all models interacting with it.
- Light gray: the metabolite is consumed by one(or more) models but also produced by one(or more) other model(s).
- Dark gray: the metabolite is only associated with one model.

To change the color code schema:

1. Mouse over the “Properties” tab to open the “PropertySheets”

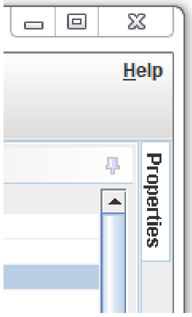

2. Scroll down to the “COMETS Plug-in”

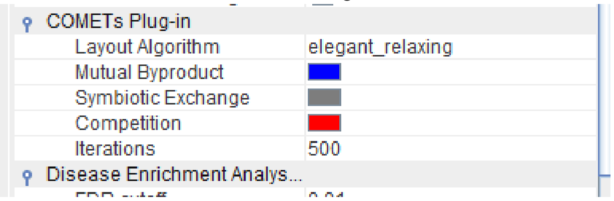

3. Use the color chooser to change the color accordingly.

Layout of your metabolic network

In VisANT 5.0, we implemented a special layout (named Symbiotic Layout) for visualization of the ecosystem level metabolic network. The new layout aims to reduce the network complexity and highlight the underlying metabolic exchanges which are biologically relevant to community dynamics. To apply the Symbiotic Layout to your network, simply click [Symbiosis] -> [Symbiotic Layout]]

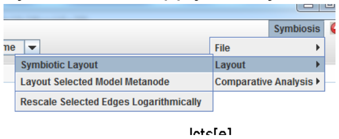

The Symbiotic Layout only works when the number of model metanodes is equal to two or greater. For each model metanode, the internal metabolites and reactions, except the exchange reactions, will form two circles inside the model. The inner circle is comprised of metabolites and the outer circle is comprised of biochemical reactions. If there are only two model metanodes in the network, two models will be placed in the same line and the environmental metabolites connected to both models will be placed in between the two model metanodes. Other extracellular metabolites are placed external to the model they are part of. If the network contains more than two model metanode, all model metanode will be arranged a large circle, while the metabolites involved in the inter-species interactions will be in the middle.

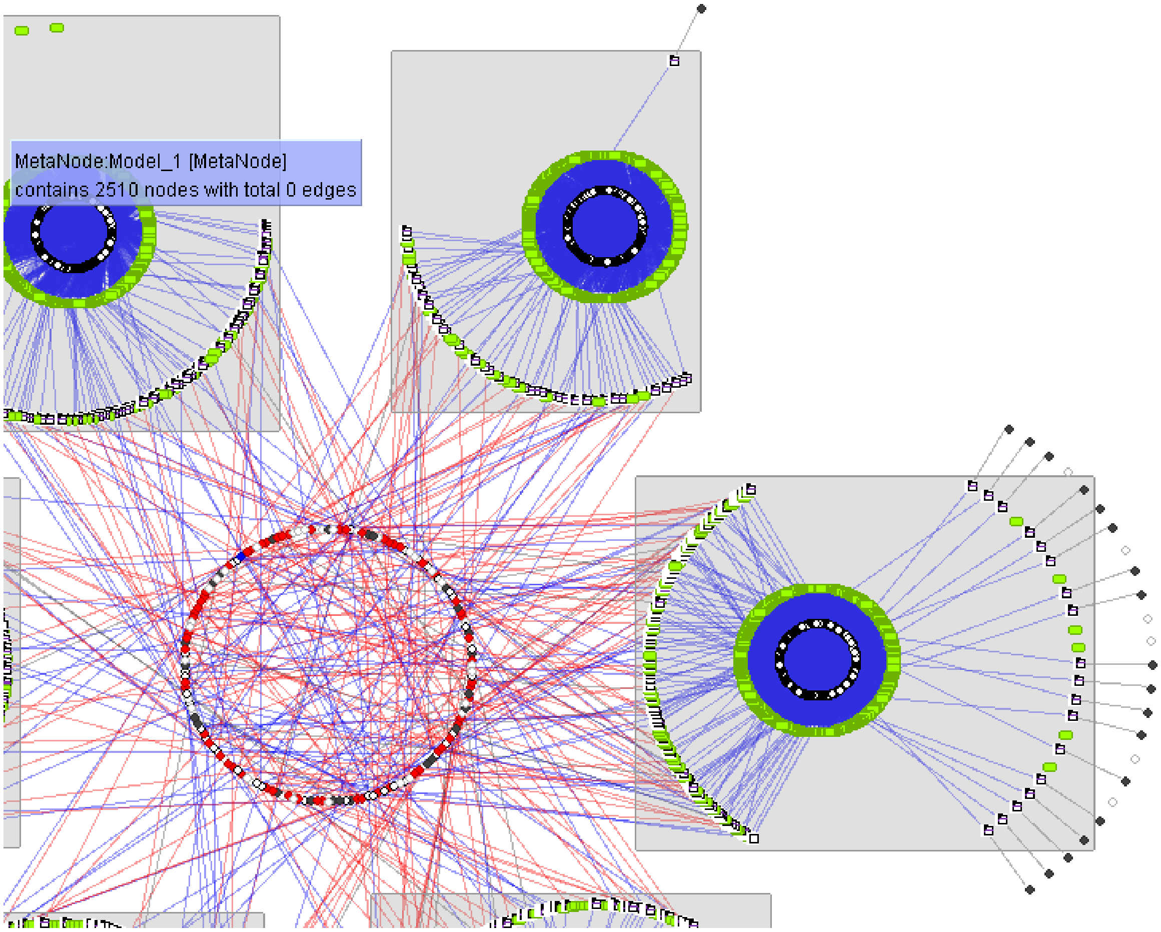

Hide/Show uninteresting metabolites

If a user supplies a list of uninteresting metabolites, a user can decide to show or hide those uninteresting metabolites from the network. For more details about the list of uninteresting metabolites, please see the "Formats related with Metabolic Networks" section. To toggle the setting

1. Open the “PropertySheets” and then scroll down to the “COMETS Plug-in”
2. Check (or uncheck) "Show Uninteresting Metabolites"

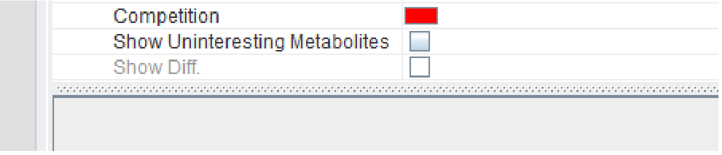

Hide/Show internal interactions

A user can decide to show or hide all internal reaction/metabolite nodes within model metanodes. To toggle the setting

1. Open the “PropertySheets” and then scroll down to the “COMETS Plug-in”
2. Check (or uncheck) "Hide Internal inter."

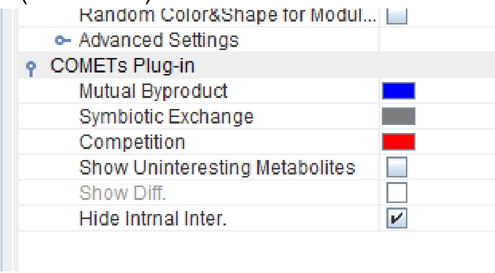

Rescale Selected Edges Logarithmically

When the flux information is available, the edge thickness will be proportional to the intensity of the flux. By default, VisANT will normalize the flux globally (namely normalize the flux distribution of the entire metabolic network). However, a user can choose to rescale the selected edges to highlight the local flux differences on the network. To rescale the selected edges

1. Select the edges which you are interested in.
2. Click [Symbiosis] -> [Layout] -> [Rescale Selected Edges Logarithmically]

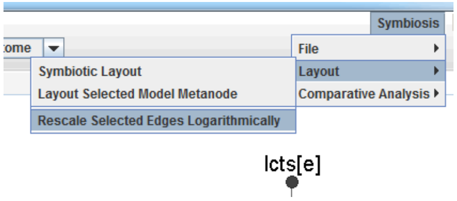

3. VisANT will rescale the flux of the selected edges (in log scale)

Notes: one way to quickly select the edge associated with the exchange metabolites is first selecting all exchange metabolites and then using the [Select Linked Nodes & Edges] in the context menu (right click menu) to select all linked edges and nodes.

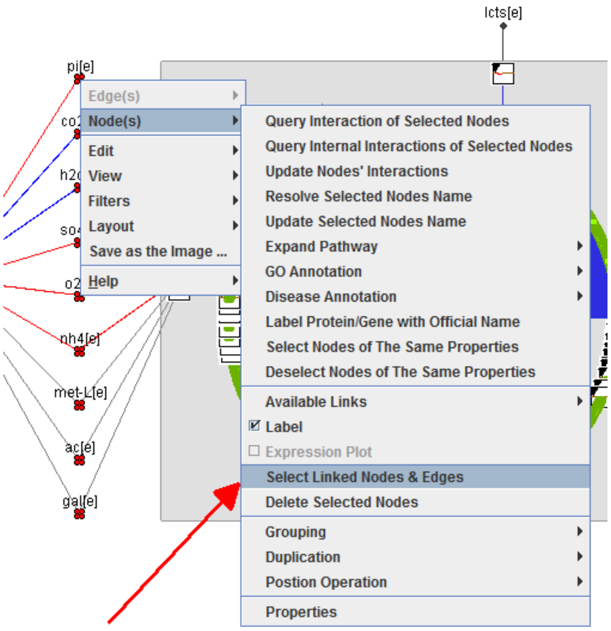

Integration functions for metabolic networks

Exploratory navigation of metabolic network

As one of the unique features of VisANT, exploratory navigation in VisANT 5.0 allows the user to walk through reactions or metabolites with step-by-step queries. Assuming that we are interested in the reactions associated with metabolite retinal (C00376), type it in the Search textfield, or using the key combination CTRL+C/CTRL+V to copy/paste, and then clicking the Search button will result in a network as shown below:

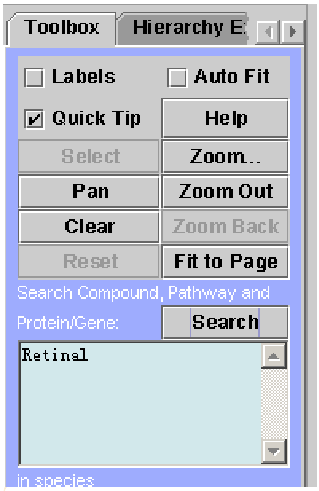

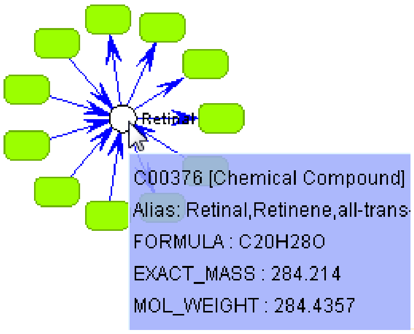

Notes: typing in KEGG compound ID C00376 will produce the same result

Notes: more information of metabolite is available on mouse-over of the node

Notes: the auto Fit option in VisANT toolbox is turned off in this example.

Notes: double click the node retinal again to toggle the hide/expand operation of reactions it involved.

Assuming the user is further interested in reaction R02128, the user can double click the reaction node to produce a network as shown below:

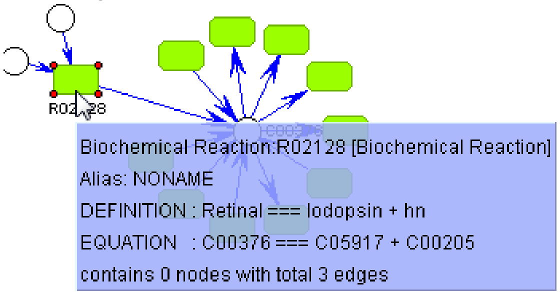

Notes: the label of R02128 is turned on for the purpoeof illustration by checking the **Label** checkbox under the menu **Nodes**

Notes: more information is available after the reaction node is queried (double-click will trigger a database query if the node has not been queried before).

Notes: same result can be achieved by using the menu **Nodes/Query Interaction of Selected Nodes** (assuming the node of interest is selected)

The exploratory navigation can continue by repeating above step for those nodes of interest. The following figure shows an example of the network after querying the labeled nodes:

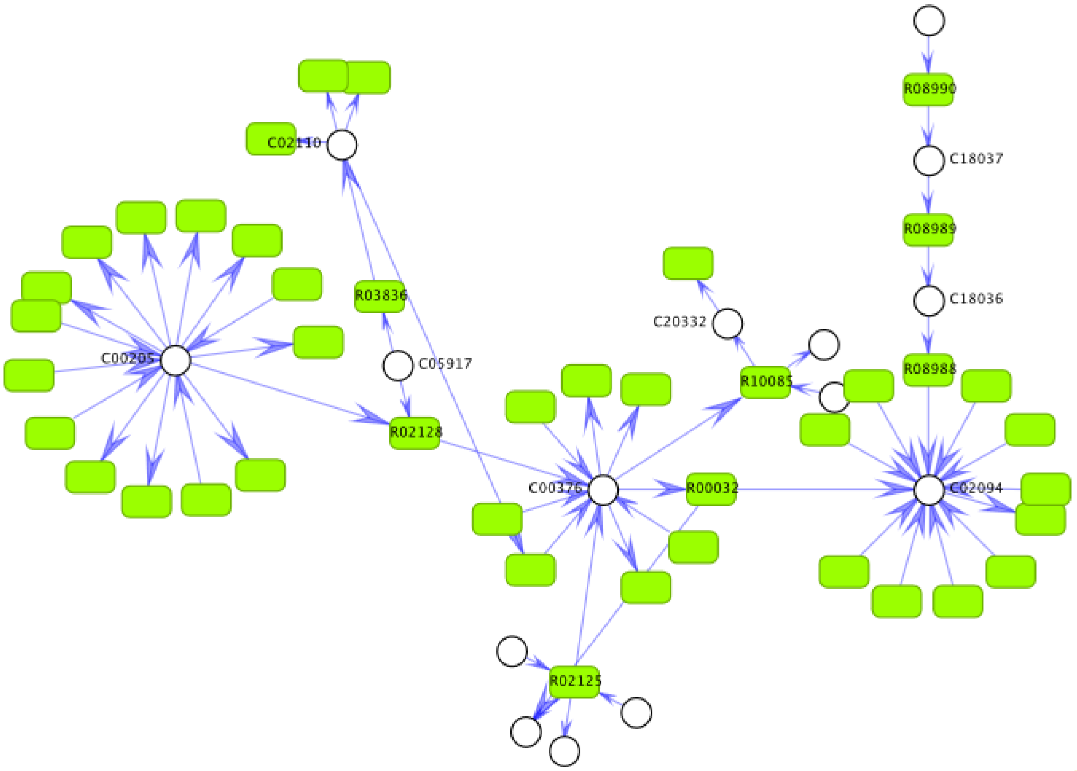

Notes: the exploratory navigation to achieve the above network can be replayed by the following macro file: http://visant.bu.edu/sample/macro/exp_nav_rcs.txt

Notes: You can load the above URL directly using the menu FIles/Open URL....if you download and run VisANT as a local application. Otherwise, you can copy/paste the content to the Add textfield and then click the Add button, as shown below:

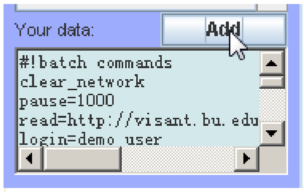

Notes: please visit http://visant.bu.edu/vmanual/cmd.htm for more information of VisANT macros.

Notes: alternatively, exploratory navigation can be started by adding nodes first:

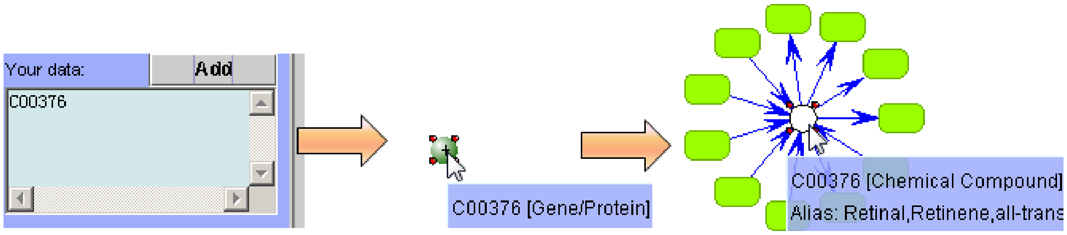

By default VisANT treats all added nodes as proteins/genes. It however will automatically recognize the right node type if the input name is unique. In this example, VisANT recognizes that C00376 is a chemical compound instead of gene/protein after querying the database.

Notes: Starting from the release of version 5.0, VisANT also supports specification of node type in the edge list format, therefore adding C00376[compound] will create a compound node in VisANT. Please visit http://visant.bu.edu/import.htm#Edge List for more information.

Integrative query of enzymes

The enzyme information of a given reaction is always Species specific. From this perspective, please make sure that the right species is selected in the VisANT toolbox. In the example below, Homo sapiens is the species of interest.

Let's first query the metabolites involved in the reaction R00623:

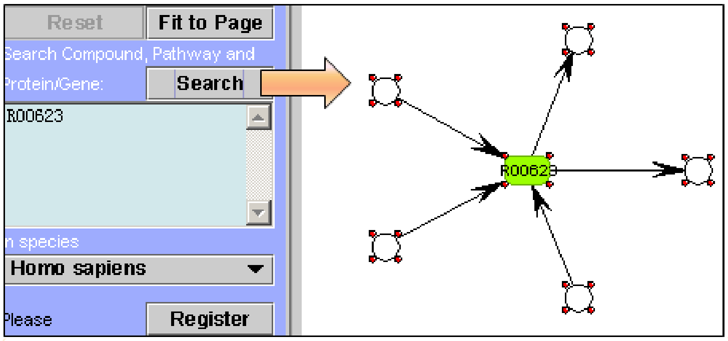

Notes: the enzyme information will not be returned when querying the interactions.

To find the enzymes of the reaction, select the reaction node(s) and use the menu **Nodes/Query Enzymes**, as shown below:

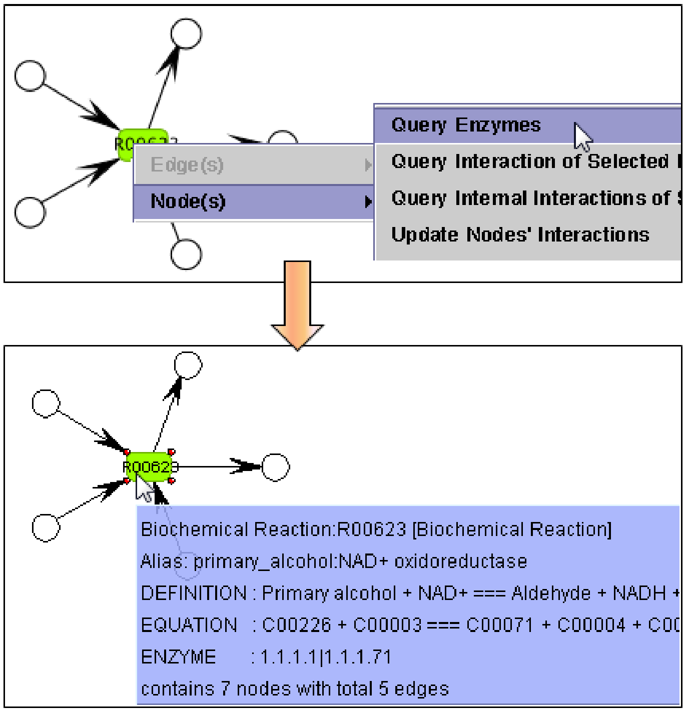

Notes: the species information is required to query the enzymes of the reaction

Notes: the **Node(s)** menu is always node specific.

There are 7 Homo sapiens enzymes for reaction R00623, as shown in the tool-tip in the above figure. The enzymes by default are embedded in the reaction node, double-clicking will expand/collapse the metanode, as shown below:

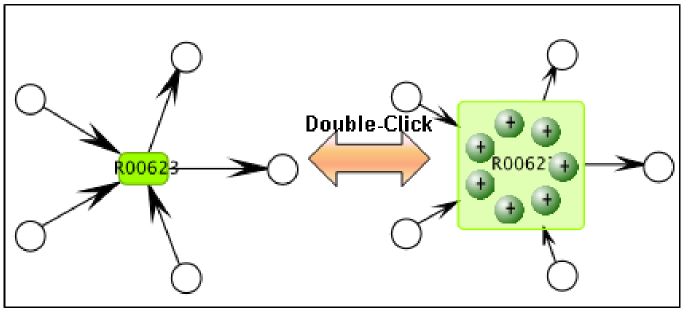

Notes: the operation of double-clicking is changed once the reaction node has the embedded enzymes. Double -clicking the reaction node with no enzyme will toggle whether associated metabolites are shown or hidden, as shown below:

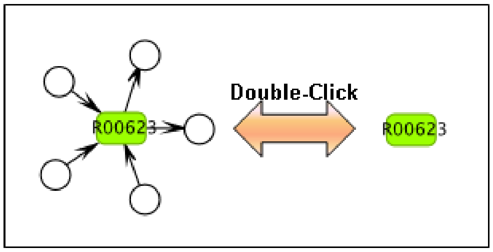

Notes: when enzymes are presented in a reaction node, expansion of the interaction of reaction node can still be performed by the menu **Node(s)/Query Interaction of Selected Node(s)**.

Notes: not all reactions have the enzyme information for a given species. Reaction nodes that have no enzyme will be turned gray after querying, as shown below:

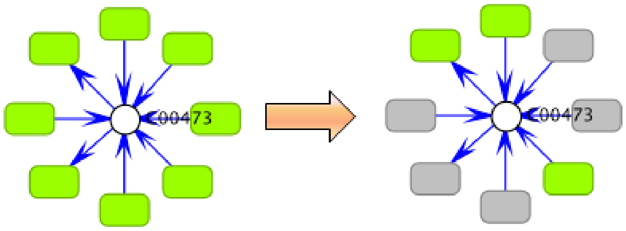

Functions associated with built-in Enzyme Nomenclature

Navigation of the hierarchy of Enzyme Nomenclature

VisANT uses the standard hierarchy tree to visualize the Enzyme Nomenclature. Clicking the expand/collapse icon will expand/collapse the EC node, as shown below:

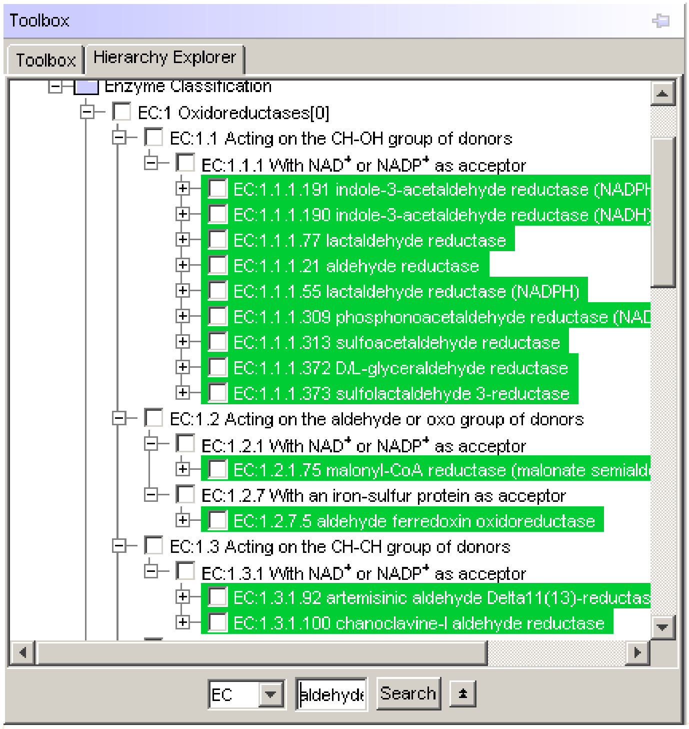

Notes: double-clicking the tree node will also toggle the expansion/collapsing of the tree node.

Notes: the width of the **Hierarchy Explorer** can be changed.

Search the Enzyme Nomenclature using the keywords

VisANT 5.0 supports keyword searching of the Enzyme Nomenclature. To perform the searching operation, first make sure the dropbox points to EC as shown in the above figure, and then type in the keywords in the search box and click the **Search** button. The figure above shows the results of the keywords “aldehyde reductase”, with corresponding tree nodes highlighted.

Drag&drop functions.

The drag&drop operations are designed to quickly create a list of reaction nodes annotated with interested enzymatic function. The following figure shows an example of dragging the EC1.6 tree node from the **Hierarchy Explorer** to the network panel where there is an existing network displaying two reactions R00281 and R10517 with corresponding metabolites:

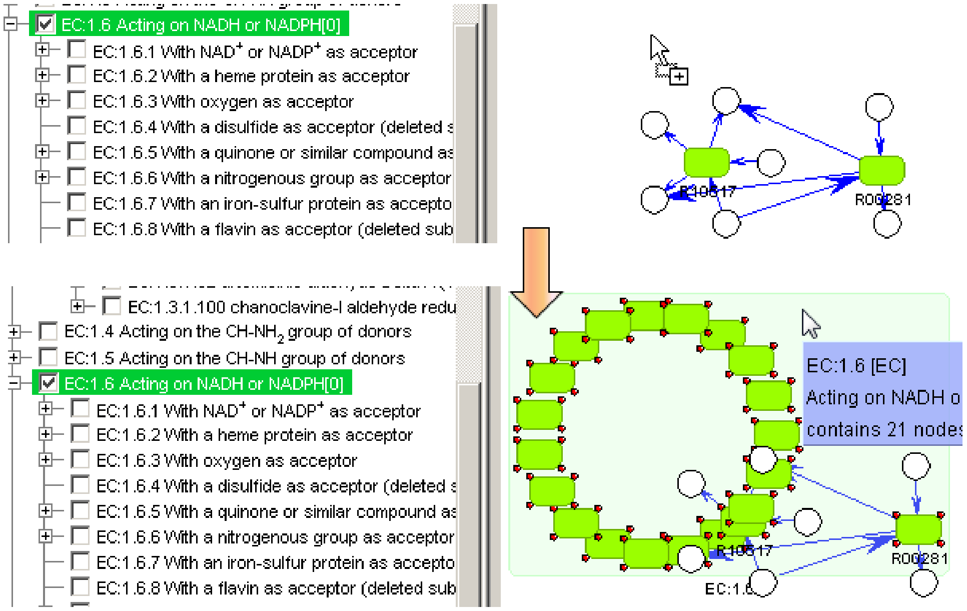

A metanode of EC 1.6 is created with total 21 reaction nodes being embedded, including R00281 and R10517.

> **Notes:** there are four total different types of hierarchical knowledge in the Hierarchy Explorer, drag&drop of tree nodes from different types of hierarchical knowledge will result in the creation of different nodes. For an example, drag&drop of a tree node from the disease hierarchy will create a metanode of disease classification embedded with genes known to be associated with this class of diseases.

> **Notes:** the combination of EC searching and drag&drop operations provides a way to search for a set of reactions using the keywords.

There are four total options for the results of the drag&drop operation, configurable by clicking the small button near the **Search** button:

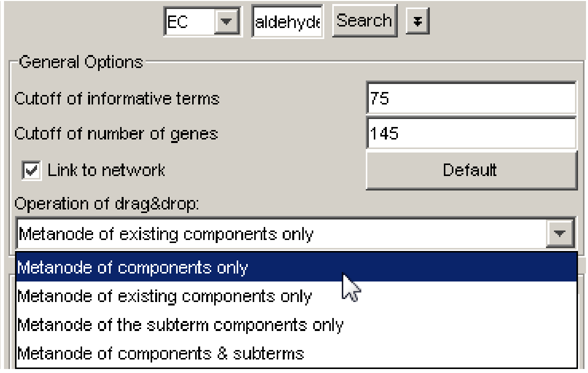

1. **Metanode of components only (default)**. This option will create an EC metanode embedded with a list of reactions annotated with the dragged tree branch
2. **Metanode of existing components only**. Same as option 1, but this option creates an EC metanode embedded with a list of reactions annotated with the dragged tree branch and already shown in the network panel. The following figure shows the resulting network when dragging the EC 1.6 tree node, using the same example as shown before:

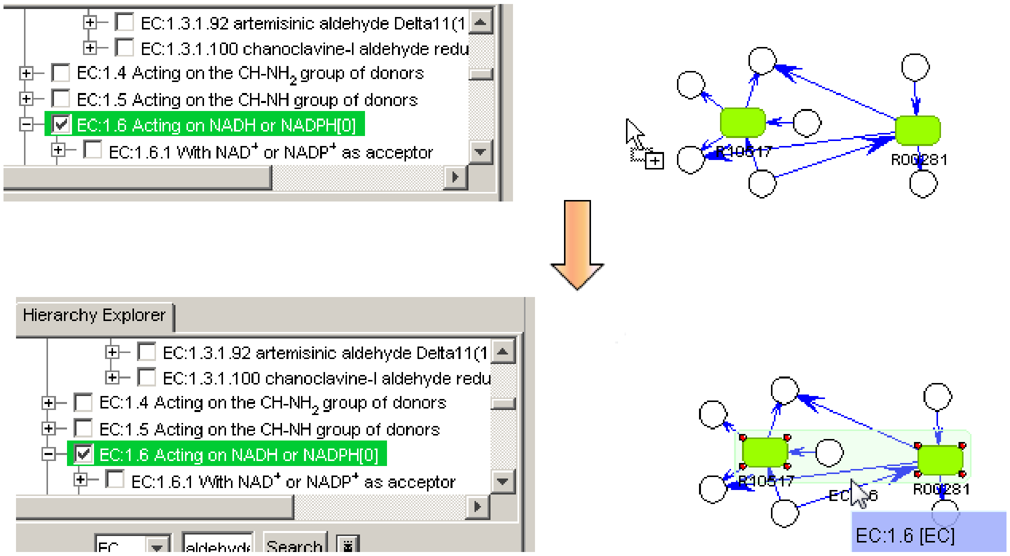

3. **Metanode of subterm components only**. This option creates a metanode embedded with reactions directly annotated under this node (be aware it is not a tree branch). In the case of EC 1.6, there is no reaction annotated directly under EC 1.6, therefore an empty metanode will be created.
4. **Metanode of components and subterms**. This option creates a metanode embedded with reactions directly annotated under this tree node, as well as all subterms under this tree node. For EC 1.6, this will create a metanode as shown below:

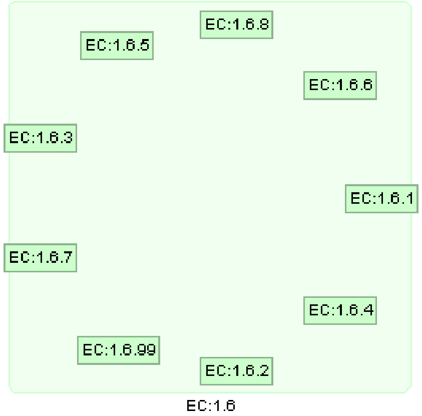

Other Miscellaneous Functions

Zooming in VisANT

- Zooming in/out can be achieved by scrolling the mouse wheel if available.
- Zooming out can be achieved by click the **Zoom Out** button on VisANT toolbox.
- Zooming in can be achieved in two steps: i) click the **Zoom In** button on VisANT toolbox; ii) use the mouse to drag a rectangular over the network region of interest to zoom in

Notes: use the **Fit to Page** button on VisANT toolbox to fit the network to the network panel in any zooming state

Notes: move the mouse with right mouse key down to pan the network

Notes: click the Select button on the VisANT toolbox to return mouse to the selection state.

Notes: zooming will not change the size of nodes whose size has been customized. Reset their size to Auto if changing the node size when zooming is preferred.

Changing the Node & Edge properties

To change the properties of nodes and edges, select nodes and edges of interest, then click the **Properties** button in the right sidebar of VisANT, and then select the corresponding properties to change, as shown below:

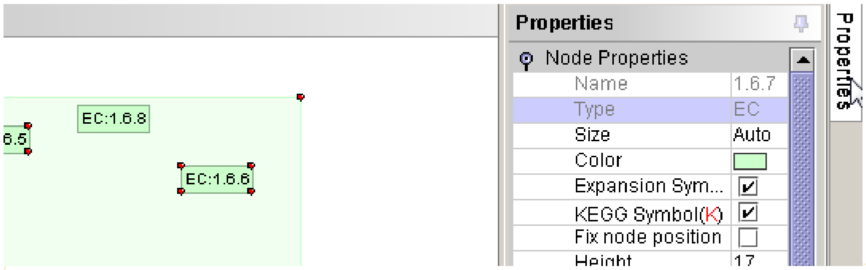

Notes: the width of the properties window can be changed if the properties window is docked (click the icon of the right-upper corner of the properties window in above figure.

Notes: some of properties of nodes and edges can also be changed using the Node(s)/Edge(s) menu for convenience purpose.

Notes: there is also some global control on VisANT toolbox to control the properties globally, such as the ability to check the **Labels** checkbox to turn on the label of all nodes.

Hide selected nodes

To hide the selected nodes, first select nodes which you want to hide and then click [Symbiosis] -> [Layout] -> [Hide Selected Nodes]. The selected node will be invisible on the network.

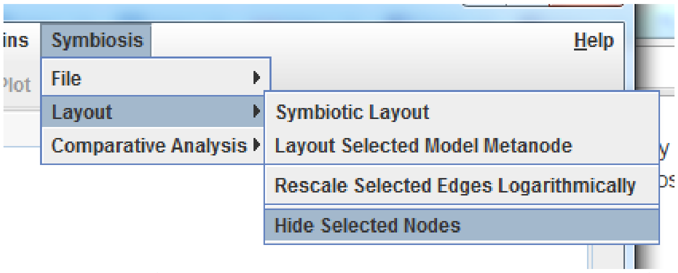

Please also reference http://visant.bu.edu/vmanual/index.html for other functions of VisANT.
