# Supplementary material for: Visualization of Metabolic Interaction Networks in Microbial Communities Using VisANT 5.0
Source: PLoS Comput Biol. 2016 Apr 15;12(4):e1004875. doi: 10.1371/journal.pcbi.1004875 (PMC4833320; doi:10.1371/journal.pcbi.1004875)
Supplement: S2 Text — (PDF) [file pcbi.1004875.s008.pdf]

Test Data and Running Instructions

Test Data 1 - the network of case study 1

The first test data set is the metabolic network of the case study 1 presented in the article. To run this example, first download the manifest file which is generated by COMETS after simulation from the following URL:

Manifest File for the Case Study 1:
http://visant.bu.edu/sample/symbio/case1/COMETS_manifest.txt

This manifest file has been modified so it will tell VisANT to fetch the rest of required files (model files, for example) from the VisANT server. For more details on the manifest file, please read the Manifest File section on page 1 of the User Manual.

From the [*Symbiosis*] →[*File*]→[*Open a manifest file...*], open the downloaded manifest file in VisANT.

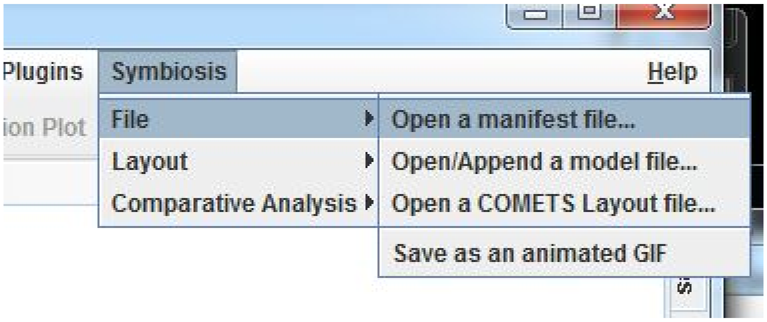

When the below dialog prompts, please select "*Load the entire world*" to visualize the metabolic activity of the whole system.

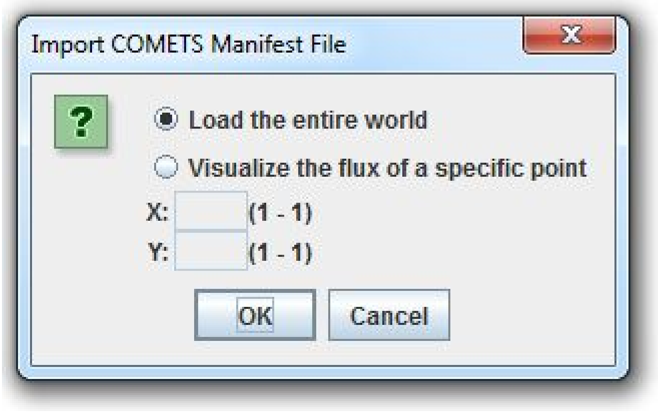

While VisANT by default will present all possible exchanges in the system at time=0, the user can use the slider to move to any other time point. VisANT will project the flux

distribution onto the network and show the underlying nutrition exchange at that particular time point.

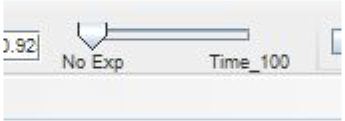

Alternatively, a user can copy and paste the following batch commands into the [*Your Data*] field in the bottom left corner and then press [*Add*] to run them.

Batch Commands

```
#!batch commands
min_edge_weight=0.00001
expression_experiement=32
symbiosis_layout
clear_selection
select_node=Model_1,Model_2
set_node_property=label_on:true
set_node_property=label_size:18
set_node_property=label_position:below
set_node_property=label_style:italic
clear_selection
select_node=met-L%5Be%5D,co2%5Be%5D,k%5Be%5D,gal%5Be%5D,ac%5Be%5D,lcts%5Be%5D,pi%5Be%5D,so4%5Be%5D,o2%5Be%5D,nh4%5Be%5D,h2o%5Be%5D
set_node_property=label_on:true
set_node_property=label_size:12
set_node_property=label_position:above
clear_selection
login=demo_user
select_node=1_R_210
query_selected_nodes
```

```
pause=150
clear_selection
select_node=1_M_1241
set_node_property=label_on:true
query_selected_nodes
pause=150
clear_selection
select_node=1_R_1620
set_node_property=label_on:true
query_selected_nodes
pause=150
clear_selection
select_node=1_M_1631
set_node_property=label_on:true
clear_selection
```

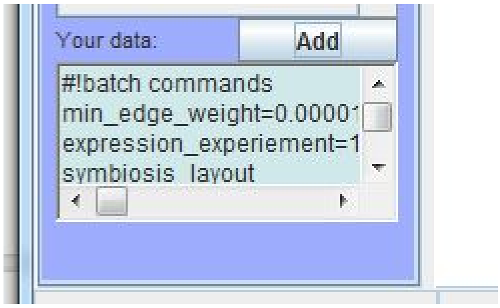

The batch commands will apply the edge weight cutoff and format the network to output a ecosystem level metabolic network as shown in the Figure 2. For more details on the VisANT batch command, please read the Batch Mode section in the VisANT user manual.

Batch Mode: http://visant.bu.edu/vmanual/cmd.htm

Test Data 2 - the network of case study 2

The second test data set is a metabolic network as shown in the Figure 3A. First download the VisANT macro file of the case study 2 from the following URL:
Case Study 2: http://visant.bu.edu/sample/symbio/case2/case_study_02.txt

Open the downloaded file in VisANT to build the metabolic interaction network in a multi-species microbial community.

The initial result will show a network after applying the symbiotic layout. Users can perform some manual rearrangements to improve the clarity and visibility of the network.

Test Data 3 - an ecosystem level network from SBML files

In test data 3, we will demonstrate how to construct a ecosystem level metabolic network from SBML files.

Users can download a manifest file of the toy example and open it in VisANT or use the '*Open URL...*' function to let VisANT directly load data from the remote server.

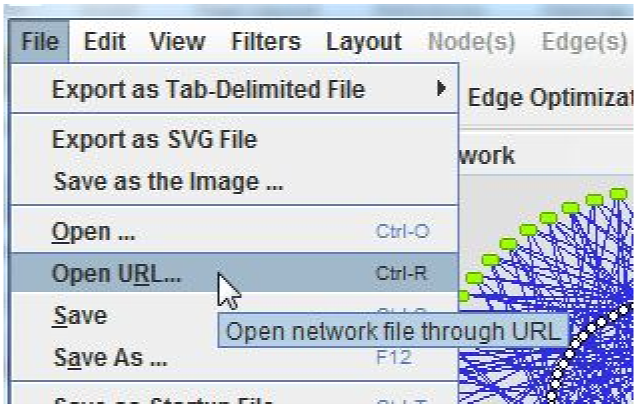

SBML Toy Example: http://visant.bu.edu/sample/symbio/manifest.txt

This toy example includes 3 metabolic models of *E. Coli* in SBML format and an example of the flux distribution in the plain text format supported by VisANT.

```
#!FBA_Manifest
ModelFileName:http://visant.bu.edu/sample/symbio/Ec_core_flux1.xml http://vi
http://visant.bu.edu/sample/symbio/Ec_core_flux1.xml
ModelLabel: Eco_model-01 Eco_model-02 Eco_model-03
FluxFileName: http://visant.bu.edu/sample/symbio/test_flux_03.txt
```
